# Evaluating the reliability of myotonometry for assessing masseter muscle hypertrophy in healthy subjects

**DOI:** 10.22514/jofph.2025.036

**Published:** 2025-06-12

**Authors:** Małgorzata Gałczyńska-Rusin, Małgorzata Pobudek-Radzikowska, Zofia Maciejewska-Szaniec, Agnieszka Przystańska, Agata Czajka-Jakubowska

**Affiliations:** ^1^Department of Orthodontics and Temporomandibular Disorders, Poznan University of Medical Sciences, 60-812 Poznan, Poland; ^2^Department of Anatomy, Poznan University of Medical Sciences, 60-781 Poznan, Poland

**Keywords:** Masseter hypertrophy, Myotonometry, Reliability

## Abstract

**Background**: Masseter muscle hypertrophy is characterized by either symmetrical or asymmetrical enlargement of the muscle, often associated with bruxism and other parafunctional habits. Traditional methods for assessing muscle hypertrophy, such as palpation and visual inspection, can be subjective and heavily dependent on the clinician’s experience. In contrast, devices like MyotonPRO offer a standardized, objective and reproducible approach, enhancing the precision and reliability of clinical diagnostics. The primary aim of our study was to evaluate the intra- and inter-rater reliability of the MyotonPRO device in assessing the viscoelastic properties of the masseter muscle. Additionally, we sought to investigate the potential correlation between subjective assessments of masseter hypertrophy and objective measurements obtained through myotonometry. **Methods**: A clinical examination using muscle palpation was conducted to identify masseter hypertrophy, categorizing participants into Normal Muscle Volume (NMV) and Muscle Hypertrophy (MH) groups. The viscoelastic properties of their masseter muscles were then measured using MyotonPRO in both relaxed and maximal contraction states. Two experienced operators performed the myotonometry on the same day, with the first operator repeating the procedure 7 days later. **Results**: Among the 58 participants, 51.7% were female, with a mean age of 28.6 years. The inter-rater reliability of masseter muscle measurements using MyotonPRO ranged from moderate to excellent, both at rest and during contraction, while intra-rater reliability ranged from moderate to good. The MH group showed higher levels of tension and stiffness, along with reduced relaxation time and creep during contraction, compared to the NMV group. The only statistically significant difference in relaxation between the groups was observed in muscle elasticity. **Conclusions**: The MyotonPRO device effectively detects statistically significant differences (*p* < 0.05) between the MH and NMV groups for certain viscoelastic parameters. However, these differences were primarily significant during contraction, with elasticity being the only parameter showing a significant difference in the relaxed state.

## 1. Introduction

The masseter muscle, one of the primary mastication muscles, plays a crucial role in various activities such as chewing, swallowing and speech [1]. The key function of this powerful muscle is to elevate the mandible, enabling mouth closure. Given its essential role, the masseter significantly influences both oral health and facial aesthetics, particularly the contour of the lower face [2]. Anatomically, the Masseter is located lateral to the ramus of the mandible and composed of three layers: superficial, deep and coronoid [3]. Moreover, its internal tendon structure subdivides the muscle into multiple partitions, which are further divided into neuromuscular compartments, delineating small motor unit territories [1].

The masseter muscle can be affected by various conditions [4, 5, 6], one of which is hypertrophy. Hypertrophy refers to an increase in muscle size due to the enlargement of individual muscle fibers rather than an increase in cell number. Generalized hypertrophy of the masticatory muscles can impact the temporalis muscles, masseters, and medial pterygoids in various configurations [5].

From a clinical point of view, masseter hypertrophy presents as either symmetric or asymmetric muscle enlargement [6]. Possible causes include bruxism [7, 8] and parafunctional habits, such as excessive gum chewing, which can increase the load on the masseter muscle and contribute to its hypertrophy [9]. Additionally, masseter hypertrophy may be congenital [10] or idiopathic [11].

Various methods can be employed to evaluate the condition of the masseter muscle. Assessment techniques include electromyography (EMG) [12], imaging techniques such as ultrasound, Magnetic Resonance Imaging (MRI) and Computed Tomography (CT) scan [5], as well as biomechanical evaluation methods like elastography and myotonometry [13, 14]. Aside from the aforementioned diagnostic tools, the masseter muscle can also be evaluated through visual inspection and palpation. Clinical diagnosis typically involves external palpation of a masseter muscle during intense clenching, along with evaluating facial asymmetry, muscle awareness and deformities in the lower third of the face.

Given that newly introduced questionnaires for bruxism evaluation now incorporate the assessments of masseter muscle hypertrophy [15, 16], this study aimed to examine the extent to which subjective evaluations of masseter palpation align with its viscoelastic properties. By analyzing the correlation between objective measurements of the masseter’s viscoelastic properties, such as stiffness, elasticity and tone, it becomes possible to validate palpation techniques. This, in turn, can help standardize evaluations, reduce variability in clinical findings, and provide a more comprehensive understanding of how hypertrophy contributes to bruxism.

Myotonometry, one of the previously mentioned methods for assessing the masseter muscles, is a non-invasive method used to evaluate muscle biomechanical properties. The MyotonPRO device (portable digital myotonometer, Myoton AS, Tallin, Estonia) is widely used in musculoskeletal research and has demonstrated reliability for assessing postural and limb muscles [14, 17, 18, 19]. However, its application in orofacial muscles, particularly the masseter, remains underexplored. While studies have validated MyotonPRO for assessing masticatory muscles, challenging factors such as muscle thickness, occlusion and parafunctional habits require further investigation.

Our research aimed to assess both intra- and inter-rater reliability using the MyotonPRO apparatus for evaluating the viscoelastic properties of the masseter muscle. Additionally, we investigated the potential correlation between subjective assessments of masseter hypertrophy and objective measurements obtained through myotonometry. We hypothesized that the stiffness, muscle tonus, elasticity, relaxation time, and creep of the masseter would differ between normal muscle volume (NMV) and muscle hypertrophy (MH) group.

## 2. Materials and methods

This study was conducted at the Poznan University of Medical Sciences between September and December 2023. A notification regarding the study was displayed on the university campus, inviting students and employees of the Medical University of Poznań to participate in the research. The inclusion criteria comprised an age range of 18–45 years, the presence of full dental arches, and providing informed consent for participation in the study. Participants were excluded if they met any of the following criteria: ongoing orthodontic treatment, neurologic and muscular disorders, Body Mass Index (BMI) >30, muscle relaxant intake, orofacial pain and painful temporomandibular disorders (TMD), severe malocclusion or asymmetries.

Painful TMD was ruled out based on the Axis I assessment conducted using the Diagnostic Criteria for Temporomandibular Disorders (DC/TMD).

### 2.1 Study design

The assessment of the masseter muscles was carried out in two stages.

In the first stage two doctors (MPR and MGR) independently assessed the structure of the muscle by palpation. Concisely, A clinical examination involving muscle palpation was utilized to identify the presence or absence of masseter hypertrophy. During the evaluation, the masseter muscle was palpated bilaterally at rest and during light clenching. The evaluation criteria included increased firmness, bulging and symmetry during light clenching. A significant increase in perceived muscle bulk and tone compared to the resting state was indicative of hypertrophy. After palpation, participants were divided into groups: Normal Muscle Volume (NMV) and Muscle Hypertrophy (MH). Subjects were included in the MH group only if both investigators independently identified the presence of masseter muscle hypertrophy. In case of disagreement between the researchers, a third researcher ZMS was asked for the final decision.

In the second stage of the study, the viscoelastic properties of the masseter muscles were assessed using the MyotonPRO device. In the resting phase, participants were instructed to relax their muscles without contact between their teeth during the examination. For the contraction phase, they were directed to clench their teeth tightly for maximum contraction. Throughout myotonometry, the patient reclined on a dentist’s chair in a supine position, and the most convex portion of the muscle belly was chosen for examination. Evaluation encompassed both the muscles on the right and left sides. The Myoton’s testing end was positioned perpendicularly on the skin surface overlying the masseter muscle.

For inter-rater reliability, each measurement point underwent assessment three times by evaluators MGR and MPR (both dentists), and the average value was calculated. Intra-rater reliability was assessed by reevaluating 12 randomly selected participants (6 from the NMV group and 6 from the MH group) after a standardized 7-day interval conducted by evaluator MGR to ensure consistency in study procedures. The general characteristics of these 12 participants for the reliability test did not significantly differ from those of the other participants in the study (*p* > 0.05). All measurements were repeated three times within the same scanning session.

The portable MyotonPRO (Myoton AS, Tallinn, Estonia) was used to measure the mechanical properties of the masseter muscles. Frequency (Hz) represents the oscillation frequency of skeletal muscle, indicating muscle tone at rest or muscle tension during contraction. Stiffness (N/m) reflects the muscle’s ability to resist changes in shape when subjected to external forces. Logarithmic decrement, measures muscle elasticity, indicating the ability to return to its original shape after contraction. During contraction, muscle elasticity increases and the logarithmic decrement decreases [17, 18]. Relaxation time (ms) or mechanical stress relaxation time, characterizes how quickly tissue recovers from displacement; higher tissue tension or stiffness leads to a faster recovery, resulting in a lower relaxation time. Creep, defined as the ratio of relaxation to deformation time, shows that tissues with higher tension, structural integrity or stiffness have greater resistance to creep, resulting in a lower value [18].

The study was approved by the University Ethics Committee under consent number 522/21. All participants provided written informed consent before participation.

### 2.2 Statistical analysis

The G*Power software (version 3.1, Heinrich-Heine-Universität Düsseldorf, Düsseldorf, NRW, Germany) was used to calculate the sample size. To determine an appropriate effect size, we referred to our previous study on the biomechanical properties of the masseter muscle in patients self-assessing for bruxism, which indicated a large effect size (ES) for stiffness (ES = 0.9) [19]. Based on this, we determined that at least 52 participants were required to achieve a power of 0.8 (1 − β error probability) with an alpha of 0.05.

Statistical analyses were performed using SPSS v23 software (IBM SPSS Statistics for Windows, Version 23.0. Armonk, NY, USA: IBM Corp). Descriptive statistics were utilized to ascertain mean values, standard deviations (SD), and minimum and maximum values of demographic variables. The normality of data distribution was assessed using the Shapiro-Wilk test. The *t*-test and Mann-Whitney U test were employed to compare differences between independent groups. A significance level of *p* < 0.05 was applied for all tests.

For inter- and intra-rater reliability the Intraclass Correlation Coefficient (ICC) was used. ICC ranges from 0 to 1. Values below 0.5 signify poor reliability, while those between 0.5 and 0.75 indicate moderate reliability. ICC values falling between 0.75 and 0.9 represent good reliability, and any value surpassing 0.9 signifies excellent reliability [20]. Parametric variables were assessed using Pearson’s correlation coefficient, while nonparametric variables were evaluated using Spearman’s rank correlation coefficient.

## 3. Results

Out of the initial 77 participants who enrolled in the study, three individuals were excluded due to undergoing orthodontic treatment, and one participant was excluded due to being diagnosed with multiple sclerosis. Subsequently, 73 volunteers underwent examination utilizing the DC/TMD questionnaire, with individuals experiencing painful TMD being excluded from the study. Ultimately, 58 participants met the criteria for assessment of the masseter muscles. The participant recruitment flow diagram is presented in Fig. 1.

**Fig. 1.** Participant recruitment flow diagram. DC/TMD: Diagnostic Criteria for Temporomandibular Disorders.

Out of the 58 participants, 51.7% were female, with a mean age of 28.6 years (SD 6.5) and a mean BMI of 22.8 (SD 3.6). Following masseter muscle palpation, the participants were categorized into two groups, *i.e.*, individuals with normal masseter volume (NMV) and those with masseter hypertrophy (MH). In one case, there was disagreement between the investigators regarding the presence of muscle hypertrophy. A third investigator was consulted to resolve the issue, and the patient was ultimately assigned to the NMV Group. None of the subjects exhibited unilateral hypertrophy of the masseter muscle mentioned above. The comparison of the groups obtained is presented in Table 1. No significant differences were found between the groups in terms of age, gender and BMI.

**Table 1.** Study group characteristics.

	Normal masseter volume n = 34	Masseter hypertrophy n = 24	*p*-value
Age (yr)	29.8 (SD 6.6)	26.8 (SD 6.1)	0.104
Sex	18 W/16 M (52.9%/47.1%)	12 W/12 M (50.0%/50.0%)	0.827
BMI	22.3 (SD 3.2)	23.5 (SD 4.1)	0.187

*Data presented as mean and standard deviation (SD). BMI: Body Mass Index; W: women; M: men.*

The inter-rater reliabilities for masseter muscle measurements using MyotonPRO ranged from moderate to excellent both at rest and during contraction. The highest ICC for inter-rater reliability was 0.937 for stiffness during contraction, while the lowest was 0.613 for decrement during contraction. The intra-rater reliabilities were rated from moderate to good. The highest ICC for intra-rater reliability was 0.887 for decrement during contraction, while the lowest was 0.710 for stiffness during contraction. The inter-rater and intra-rater reliability values for the ICC are displayed in Table 2. Inter-rater reliabilities were lower at rest than during contraction, except for decrement. Conversely, intra-rater reliabilities were lower during contraction than at rest, also except for decrement.

**Table 2.** The inter-rater and intra-rater reliability values.

MyotonPRO parameters	Inter-rater		Intra-rater	
	ICC	95% CI	ICC	95% CI
Frequency R	0.797	0.439–0.900	0.835	0.703–0.908
Frequency C	0.908	0.823–0.951	0.763	0.574–0.868
Stiffness R	0.820	0.629–0.907	0.876	0.775–0.931
Stiffness C	0.937	0.884–0.966	0.710	0.481–0.839
Decrement R	0.854	0.736–0.919	0.854	0.724–0.921
Decrement C	0.613	0.297–0.787	0.887	0.792–0.938
Relaxation time R	0.775	0.448–0.894	0.854	0.737–0.919
Relaxation time C	0.893	0.792–0.943	0.777	0.597–0.877
Creep R	0.766	0.421–0.890	0.847	0.726–0.915
Creep C	0.884	0.777–0.938	0.789	0.620–0.883

*Abbreviation: R: resting; C: contraction; CI: confidence interval; ICC: interclass correlation coefficient.*

The next stage of the study involved assessing the viscoelastic properties of masticatory muscles. Table 3 displays the specific viscoelastic properties of the masseter muscles, categorized by the right and left sides masseter of the groups under investigation. Upon palpation, participants with masseter hypertrophy exhibited notably higher levels of tension and stiffness, as well as lower levels of relaxation time, and creep during contraction compared to the normal volume muscle group. Regarding relaxation, the only statistically significant difference between the groups was observed in muscle elasticity.

**Table 3.** Statistical comparison MyotonPRO® of tone/tension, stiffness, elasticity, relaxation time and creep between patients with normal masseter volume and masseter hypertrophy.

Parameter	Condition	Normal Masseter Volume (Mean ± SD)	Hypertrophy (Mean ± SD)	*p*-value
Frequency (Hz)				
	Right relaxed	15.7 ± 4.1	14.7 ± 2.2	0.239
	Left relaxed	15.8 ± 4.1	14.5 ± 2.0	0.123
	Right contracted	18.2 ± 3.2	20.8 ± 3.1	**0.003**
	Left contracted	19.0 ± 3.5	20.8 ± 3.6	0.064
Stiffness (N/m)				
	Right relaxed	330.2 ± 93.5	299.2 ± 68.6	0.151
	Left relaxed	324.4 ± 93.1	290.2 ± 57.5	0.090
	Right contracted	413.0 ± 125.1	555.6 ± 161.4	**0.001**
	Left contracted	442.9 ± 146.3	541.6 ± 141.4	**0.013**
Elasticity (Decrement)				
	Right relaxed	1.95 ± 0.36	1.78 ± 0.21	**0.023**
	Left relaxed	1.93 ± 0.39	1.75 ± 0.23	**0.039**
	Right contracted	1.51 ± 0.41	1.43 ± 0.28	0.382
	Left contracted	1.51 ± 0.39	1.44 ± 0.37	0.518
Relaxation (ms)				
	Right relaxed	18.4 ± 4.5	20.1 ± 4.0	0.148
	Left relaxed	18.3 ± 4.5	20.4 ± 3.7	0.062
	Right contracted	13.7 ± 4.4	9.7 ± 3.2	**<0.001**
	Left contracted	12.6 ± 3.6	10.3 ± 3.7	**0.023**
Creep				
	Right relaxed	1.15 ± 0.26	1.25 ± 0.25	0.151
	Left relaxed	1.14 ± 0.27	1.28 ± 0.23	0.055
	Right contracted	0.85 ± 0.25	0.63 ± 0.18	**0.001**
	Left contracted	0.79 ± 0.39	0.66 ± 0.22	**0.034**

*Significant results (p < 0.05) are highlighted in bold. T-test was performed unless otherwise indicated. Data presented as mean and standard deviation (SD).*

Crucially, no statistically significant differences were found between the measured parameters of the right and left masseter muscles.

## 4. Discussion

Masseter muscle hypertrophy has been incorporated as a parameter in new bruxism evaluation questionnaires. According to the Stab questionnaire, the examiner is required to identify any obvious hypertrophy of the masseter muscle where the muscle size exceeds the expected size of the patient’s face [16]. However, the assessment method, whether visual or palpatory, and whether the muscle should be evaluated during contraction or relaxation are not specified. Similarly, the BruxScreen questionnaire also assesses the presence or absence of masseter muscle hypertrophy. To that end, the dentist observes the masseter muscles in two conditions: at rest and during contraction [15]. Currently, there are no precise guidelines in the literature for assessing the masseter hypertrophy in bruxers. However, scales have been created for the needs of aesthetic medicine. Xie *et al*. [21] classified the masseter muscle based on the type of its bulge, depending on the contraction and thickness of the muscle. They distinguished 5 types (minimal, mono, double, triple, excessive). Based on the above classification and assessment of the muscle thickness, the authors determined the appropriate dose and number of injections for the administration of botulinum toxin type A [21]. Han *et al*. [22] developed the masseter muscle hypertrophy grading scale to evaluate the overall hypertrophy degree, with grades ranging from 1 (minimal) to 5 (very marked). Both scales were designed based on Asian populations.

Given the subjective nature of the masseter hypertrophy assessment, which relies on visual inspection and palpation, this study aimed to determine the extent to which subjective evaluation aligns with objective measurements obtained using the MyotonPRO device.

Previous studies have demonstrated that myotonometry exhibits good to excellent reliability, validity, and precision for diagnostic purposes across diverse patient populations [14, 23, 24]. This increasingly popular method is used to assess not only muscles but also tendons and the skin surface [25, 26, 27]. In Song *et al*. [24] study, which focused solely on stiffness in the masticatory muscle, both intra-operator and inter-operator reliability exceeded 0.98. Moreover, Taş *et al*. [28] examined the masticatory muscle in a relaxed state and observed the highest inter-rater reliability for decrement and creep (0.82), while the lowest for frequency (0.72). Furthermore, for intra-rater reliability, the highest value was recorded for decrement (0.88) and the lowest for relaxation time (0.66) [28]. In our study, only the inter-rater reliability for decrement during contraction (0.61) and the intra-rater reliability for stiffness during contraction (0.71) showed moderate ICC values (<0.75). For all other parameters tested, the ICC values ranged from good to excellent, demonstrating that myotonometry is a reliable method for assessing the viscoelastic properties of the masticatory muscles. However, to ensure accurate measurements, it is essential to carefully consider factors that influence intra-rater variability.

To the best of the authors’ knowledge, this is the first study to evaluate all MyotonPRO parameters (frequency, stiffness, decrement, relaxation time, creep) while assessing both inter- and intra-rater reliability of the masseter muscles in both relaxed and contracted states.

In our study, significant differences in masseter muscle tension were observed between the NMV and MH groups. Muscle tension measurement, based on the acceleration signal’s natural frequency, provides valuable insight into the muscle’s intrinsic properties. According to Gavronski *et al*. [29] a higher oscillation frequency corresponds to greater muscle tension, which increases with contraction. In the masseter hypertrophy group, frequency values were significantly higher during contraction. Interestingly, the MH group exhibited lower frequency values at rest than the NMV group, though this difference was not statistically significant.

Muscle stiffness is the most commonly reported parameter in studies utilizing MyotonPRO. It reflects the tissue’s resistance to external forces that alter its shape, with higher stiffness indicating a greater energy requirement for such deformation. As muscle contracts, its stiffness increases proportionately to the force of contraction [30]. In our study, stiffness increased during masseter contraction, aligning with findings from other researchers [29, 31, 32]. Gavronski *et al*. [29] similarly observed that skeletal muscle stiffness values are higher during contraction than relaxation. Moreover, Mustalampi also noted that muscle stiffness progressively increases with greater force production [31]. The higher the N/m value, the stiffer the muscle and the less it relaxes [29]. Our study observed a difference in stiffness during muscle contraction between the NMV and MH groups. We found that the stiffness of contracted hypertrophied muscle is significantly higher, indicating that more force may be needed to stretch its muscle via its antagonistic muscles. Mackala *et al*. [33] suggest that increased muscle stiffness can negatively affect microcirculation, which in turn reduces the muscle’s ability to support exercise.

The muscle’s elasticity is defined by the logarithmic decrement of its natural oscillation, reflecting its capability to regain its original shape post-deformation [18]. A reduced decrement value indicates less dissipation of mechanical energy and superior muscle elasticity [29, 32]. Reduced elasticity indicates increased movement difficulty and a higher tendency for fatigue [17]. Our research reveals a significant disparity in muscle elasticity at rest between NMV and MH. This discrepancy represents the sole significant difference noted in muscle relaxation between the aforementioned groups. A lower value signifies better muscle tissue elasticity, requiring less energy for change. Typically, muscle tissue elasticity increases during contraction [17]. Moreover, during contraction, the elasticity of the masseter increased, as shown by a decrease in decrement. However, no significant disparity was observed between the NMV and MH groups. Some researchers caution against assessing elasticity. Fröhlich-Zwahlen *et al*. [34], for instance, do not recommend elasticity evaluation due to concerns about reliability. Similarly, Mustalampi *et al*. [31] argue that the oscillation decrement parameter has not proven to be a reliable indicator for detecting clinically relevant muscle changes. However, Gavronski *et al*. [29] assert that muscle elasticity increases during contraction, potentially mitigating injuries. Furthermore, they suggest that elasticity could be a quality derived from the functional properties of muscles, as specific skeletal muscles retain their elasticity even during relaxation.

The last two values: relaxation time and creep were significantly lower during contraction for the MH group. Relaxation time refers to the duration required for a muscle to return to its resting shape after being deformed. Creep is calculated as the ratio of relaxation time to deformation time, with deformation time being the interval needed for the myotonometer’s testing probe to penetrate the tissue fully using a consistent force [18, 35]. According to Mencel *et al*. [35], lower values for relaxation time and creep indicate higher muscle tension or stiffness. This is consistent with our observations. In contrast, Della Posta *et al*.’s [36] findings suggest that prolonged relaxation time may be associated with muscle dysfunction.

No statistically significant differences were observed in any measured parameters between the right and left sides, either in a relaxed state or under maximum bite force. This could be attributed to the absence of patients with unilateral masseter muscle hypertrophy in our cohort. Nonetheless, this finding aligns with Yu *et al*.’s [14] observations, which suggest that both sides of the masseter muscle are typically equally engaged in supporting masticatory function physiologically .

## 5. Limitations

The study focused on patients without painful symptoms in the masticatory muscles. Future research should include a larger sample size, incorporating patients with painful forms of TMD. Additionally, this study did not account for the presence of parafunctional habits or habitual chewing side preference, both of which may contribute to the development of muscle hypertrophy. Another limitation of this study is the lack of structural imaging techniques, such as ultrasonography or MRI, to assess the morphology of the masseter muscle. Integrating structural measurements, such as muscle thickness or cross-sectional area, with the viscoelastic properties measured in this study could provide a more comprehensive understanding of the relationship between muscle structure and function. Future research should incorporate these imaging modalities to enhance the analysis and explore the interplay between structural and biomechanical characteristics of the masseter muscle in greater detail. Although myotonometry is gaining popularity as a diagnostic tool, especially in the assessment of orofacial muscles [36, 37, 38], large-scale studies are necessary to establish normal reference ranges for comparing patients with muscle abnormalities. For this reason, clinicians should use it as a supplementary rather than a stand-alone diagnostic tool.

## 6. Conclusions

The MyotonPRO device is a reliable tool for detecting a statistically significant difference (*p* < 0.05) between the MH and an NMV group for certain viscoelastic parameters. However, these differences were predominantly significant for most parameters in contraction, with elasticity being the only parameter that showed a significant difference in the relaxed state. To the authors’ knowledge, this is the first study to examine the discriminant validity of the Myoton myotonometer by investigating the masseter muscle in different states.
